# Clinical Implications of Dietary Probiotic Supplement (Associated with L-Glutamine and Biotin) in Ulcerative Colitis Patients’ Body Composition and Quality of Life

**DOI:** 10.3390/nu15245049

**Published:** 2023-12-08

**Authors:** Flavia Maria Pavel, Simona Gabriela Bungau, Delia Mirela Tit, Timea Claudia Ghitea, Ruxandra Cristina Marin, Andrei-Flavius Radu, Radu Dumitru Moleriu, Tiberia Ilias, Cristian Bustea, Cosmin Mihai Vesa

**Affiliations:** 1 Doctoral School of Biological and Biomedical Sciences, University of Oradea, 410087 Oradea, Romania; flavia.bontze@gmail.com (F.M.P.); andreiflavius.radu@uoradea.ro (A.-F.R.); cosmin.vesa@csud.uoradea.ro (C.M.V.); 2 Department of Preclinical Disciplines, Faculty of Medicine and Pharmacy of Oradea, University of Oradea, 410073 Oradea, Romania; 3 Department of Pharmacy, Faculty of Medicine and Pharmacy, University of Oradea, 410028 Oradea, Romania; timea.ghitea@csud.uoradea.ro; 4 Fundeni Clinical Hospital, 022328 Bucharest, Romania; rcfhm@yahoo.com; 5 Department of Mathematics, Faculty of Mathematics and Computer Science, West University of Timisoara, 300223 Timisoara, Romania; radu.moleriu@e-uvt.ro; 6 Department of Medical Disciplines, Faculty of Medicine and Pharmacy, University of Oradea, 410073 Oradea, Romania; tiberia_ilias@yahoo.com; 7 Department of Surgery, Oradea County Emergency Clinical Hospital, 410169 Oradea, Romania; cristibustea@yahoo.com

**Keywords:** inflammatory bowel diseases, ulcerative colitis, dysbiosis, body composition, quality of life, probiotic

## Abstract

Patients with ulcerative colitis (UC) are reported to have changes in body structure, with negative impact on the course of disease. This study explored the effects of a standardized nutritional supplement containing five bacterial strains of at least five billion bacteria (*Bifidobacterium infantis*, *Bifidobacterium animalis*, *Lactobacillus bulgaricus*, *Lactobacillus helveticus*, and *Enterococcus faecium)*, L-glutamine, and biotin on the body composition and quality of life of patients with UC. Ninety-three patients over 18 years of age with a confirmed diagnosis of UC, for whom body composition could be accurately determined, were included in this observational follow-up randomized study. These patients were split into two groups: UC-P (44 patients with dietary counselling and supplement with probiotics) and UC-NP (49 patients with dietary counselling, without supplement). Body composition was assessed using the multifrequency bioelectrical impedance device, and the quality of life related to UC was evaluated by applying the short inflammatory bowel disease questionnaire (SIBDQ). The results showed that the average value of muscular mass (MM) and sarcopenic index (SMI) significantly increased (*p* = 0.043, respectively, *p* = 0.001) and a large fraction (*p* = 0.001) of patients had their SMI levels normalized in the UC-P group compared with UC-NP group. The extracellular water to total body water ratio (ECW/TBW) also had significantly different mean values (*p* = 0.022), favoring the UC-P group. By testing the differences between the average values of body composition parameters before and after treatment, we obtained significant results in body mass index (BMI) (*p* = 0.046), fat free mass (FFM) (*p* < 0.001), and ECW/TBW ratio (*p* = 0.048). The SIBDQ total score increased significantly (*p* < 0.001) in the UC-P group and was more strongly associated with changes in body parameters. Supplementation with probiotics associated with L-glutamine and biotin can improve body composition parameters, which in turn implies an increase in the overall quality of life of patients with UC.

## 1. Introduction

The prevalence of inflammatory bowel disease (IBD), including UC, is continuously rising and has emerged as a significant public health concern [1]. Patients with IBD are reported to have changes in body structure, with these disruptions having a negative impact on the quality of life, treatment response, and other IBD-related side effects [2].

Ulcerative colitis (UC) is an inflammatory condition that affects the colonic mucosa or the rectum. It is a chronic, diffuse, and incurable disease. It is the most prevalent type of inflammatory bowel disease (IBD). Genetic susceptibility, epithelial barrier abnormalities, dysregulated immunological responses, and environmental variables all play a role in the etiology [3,4]. Although UC incidence is rising gradually everywhere, with the highest rates observed in northern Europe, Canada, and Australia, it is regarded as a global disease [5,6]. 

Chronic inflammation can alter the homeostasis of muscles, bones, and fat, as well as contribute to the emergence of malnutrition and abnormal body composition, many times being associated with cancer [7]. Decreased skeletal muscle and fat volumes can be brought on by increased energy expenditure brought on by inflammation, compromised digestion and absorption, protein leakage brought on by ulcerative lesions, and other factors [8]. In patients with UC, altered body composition, such as decreased fat-free mass, has been noted. Inadequate intake, poor absorption, nutritional loss, and increased demand for nutrients all contribute to these changes. A poor prognosis, malnutrition, reduced muscle mass, and sarcopenia [9] can result from these alterations in body composition [10,11].

Patients’ quality of life is influenced by their nutritional status. Nevertheless, there is no accurate method for determining nutritional status (NS) in this circumstance. For a patient’s nutritional evaluation, a body composition analysis is essential. In inflammatory bowel disease (IBD), body composition is analyzed using a variety of methods, each with unique properties, such as dual X-ray absorptiometry (DXA), computed tomography (CT) or magnetic resonance imaging (MRI), and body image analysis (BIA) [12]. Although DXA has long been considered the “gold standard” for determining body composition, studies have also highlighted the accuracy and consistency of determining body composition using CT and MRI scans. However, these procedures are typically inaccessible due to their high cost, risk of radiation exposure (CT), and frequency [13]. Typically, studies have used bioelectrical impedance analysis (BIA) to examine the NS of IBD patients, as a portable, easy-to-use, inexpensive, non-invasive, and safe technique for assessing body composition [14]. Measuring both fat mass (FM) and fat-free mass (FFM) is necessary to assess nutritional status in individuals with IBD/UC [15]. The phase angle (PhA) determined from BIA is a variable that can be calculated from the connection between resistance (R) and reactance (Xc) (R/Xc 180°) and appears to be a new and promising indication of nutritional status. Although its biological significance is not fully known, it is considered to be a sign of membrane resiliency and water distribution between intracellular and extracellular compartments [16]. Each compartment’s water fraction could disclose details about the body’s health. The ratio of extracellular water to total body water (ECW/TBW) is a reliable marker of edema in patients and it is highly correlated with the severity of nutritional condition [17,18]. Serum albumin and hemoglobin levels, as well as the duration spent under mechanical ventilation, were found to be inversely correlated with the ECW/TBW ratio [19]. 

UC treatment is based on the intensity, distribution, and nature of the illness, as well as its course, the patient’s reaction to earlier drugs and any adverse effects, the likelihood of relapse, and extra intestinal symptoms. Achieving clinical remission that is verified by endoscopic testing is the main objective of treatment [20]. Steroids, immunosuppressant, 5-aminosalicylic acid, and biologic agents are all used to treat ulcerative colitis [17,21]. Although the therapeutic options for this pathology have diversified in recent years, many patients failed to obtain remission after treatment or experienced a gradual loss of response to it. In addition, a significant percentage of patients experienced specific gastrointestinal symptoms, accompanied by severe fatigue, even during periods of remission [22,23]. Because of these drawbacks, as well as the high cost of classical therapeutic agents, the search for alternatives with therapeutic or adjuvant potential in UC is an ongoing concern for clinicians and health researchers. Due to the high anti-inflammatory and immunomodulatory potential and the ability to correct intestinal dysbiosis [24,25,26] (which are specific in UC), probiotics have been considered a therapeutic option for patients with this condition. Probiotics, particularly those belonging to the *Lactobacillus*/*Bifidobacterium* sp., have been shown to be helpful in the treatment of UC. The primary demonstrated advantages of probiotics for UC patients are the decrease in clinical signs and symptoms, as well as the induction and maintenance of the remission period [27]. L-glutamine is a “conditionally essential” amino acid that prevents damage to the gastrointestinal tract and restores lumen barrier failure. As a result, supplementing probiotics with L-glutamine lowers clinical and endoscopic scores in UC patients while promoting gastrointestinal mucosal development and limiting permeability to toxins and pathogens, atrophy mucosa, and villi [28]. 

UC has also been linked to biotin deficiency, a water-soluble essential B vitamin and a crucial component in several metabolic pathways [29]. Its conventional function is as a covalently bound coenzyme for carboxylases used in leucine catabolism, gluconeogenesis, branched-chain amino acid and odd-chain fatty acid breakdown, and fatty acid synthesis and oxidation [30]. More recently, its noncarboxylic biological functions have also come to light, including those related to cell signaling, epigenetic gene control, chromatin structure, and immunological response [31,32]. New research on biotin’s function in the immune system showed that it is associated with inflammation and that a biotin shortage increases the production of pro-inflammatory cytokines [30]. Moreover, recent studies indicated that a biotin deficit leads to intestinal dysbiosis, one of the primary causes of IBD [29].

By selecting a dietary supplement in which probiotics are associated with glutamine and biotin, our investigation aimed to assess the clinical effects of this particular supplement on body composition and quality of life in UC patients. This is a novel approach in the field of therapies in UC.

## 2. Results 

This study included 93 adult UC patients with an average age of 46.62 ± 16.13 years. The ratio of men to women was 1:1.11. Initially, there were no significant differences between the two groups in terms of the demographics and clinical characteristics, as described in Table 1.

The examination of BMI and body composition revealed that at the initial evaluation there were no statistically significant differences between the two groups (*p* > 0.05) in the studied variables; at the end of the research, the outcomes showed an improvement, particularly in the group of patients receiving probiotics. MM, SMI, and ECW/TBW showed significant rises or decreases (*p* < 0.05) (Table 2), demonstrating positive impacts of the probiotics -based strategy. 

To predict the evolution of the data, a Wilcoxon signed rank test was applied to both patient groups at different points in time (the start and the end of the study), with the UC-P group achieving better results (Table 3). Although there was an improvement in the UC-NP group where patients received specialized nutritional counseling only, the outcomes were not as good as the outcomes in the UC-P group.

**Figure 1:**
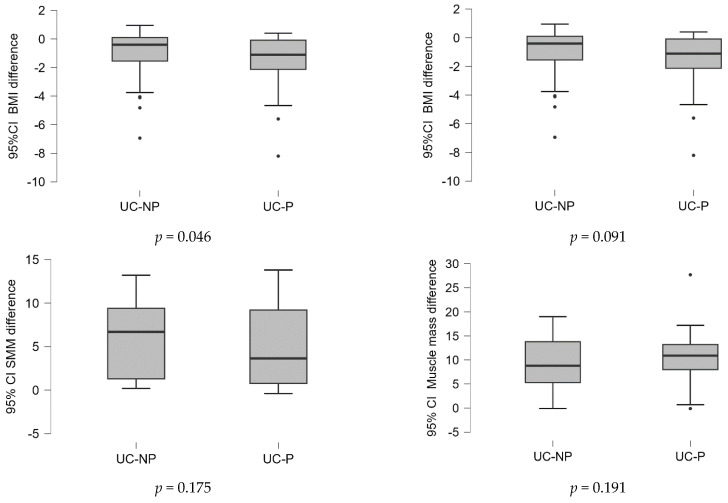

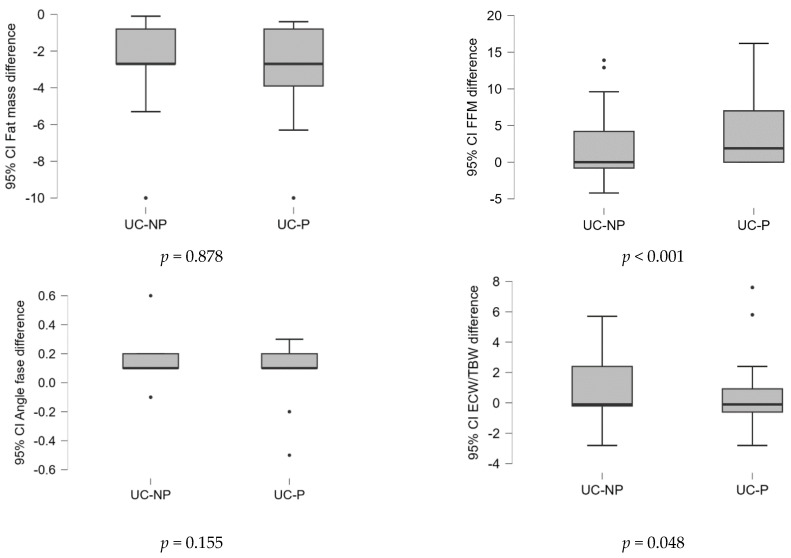
Differences of the values of variables in the two time points (before/after the treatment) analyzed between samples. UC-P—group with dietary counselling and supplement with probiotics, L-glutamine, and biotin, UC-NP—group with dietary counselling, without supplement.

Considering that the data were not initially completely homogeneous, the differences in the values of the variables at the two time points (before/after the treatment) were calculated and examined between samples. Based on the statistical tests performed, the results were graphically represented, as shown in Figure 1. Because the data were not normally distributed, the Mann–Whitney U nonparametrically test was used. The findings shown in Figure 1 demonstrate that when BMI, FFM, and PA were calculated and compared between samples, there was a significant statistical difference (*p* < 0.05). 

To highlight the differences between the two sides’ final outcomes, an analysis of the body composition parameter results and the evolution of BMI in relation to reference values was conducted. At the time of the initial assessment, the proportion of overweight patients was considerably greater in the UC-P group (*p* < 0.001), but by the time the study was complete, the difference had reversed, with the proportion of normal-weight patients in this group becoming significantly higher than in the control group (*p* < 0.001). When the body composition parameters were examined, the results showed an improvement in the levels of SMI, FM, and AP, where a significant drop in the percentage of patients (*p* < 0.05) with values outside the reference limits had been noted. Probiotic use had proven to be a significant protective factor in the evolution of the evaluated parameters. 

Also, the significant decreases in the proportion of patients with CRP > 10 mg/L values in the UC-P group, as compared to the UC-NP group, showed the supplement’s anti-inflammatory benefits (Table 4).

### Evolution of SIBDQ Score

At baseline evaluation, the mean values of the total SIBDQ score showed a moderate to severe decline in life quality (<60) in both groups, without significant differences (45.21 in UC-P group vs. 45.58 in UC-P group, *p* = 0.061). 

The means for the total score were significantly larger at the end of the study in the group with probiotics, compared to the control group (60.47 vs. 51.05, *p* <  0.001) (Table 5). The study of the SBDIQ’s domains revealed that the UC-P group had considerably improved evolution, compared to the UC-NP group (all *p* < 0.001). Table 5 displayed the mean domain and overall SIBDQ scores at baseline and end evaluation. 

By applying the Wilcoxon signed rank test, a significant increase (*p* < 0.05) in the total and domain scores was observed in both groups, with a better evolution in the UC-P group (Table 6). In the UC-P group, the average SIBDQ total score increased slightly above 60 (60.47) at the final evaluation, which indicated a mild impairment of the quality of life, while in the UC-NP group this score remained below 60 (51.05), indicating a moderate impact on the quality of life of these patients.

**Figure 2:**
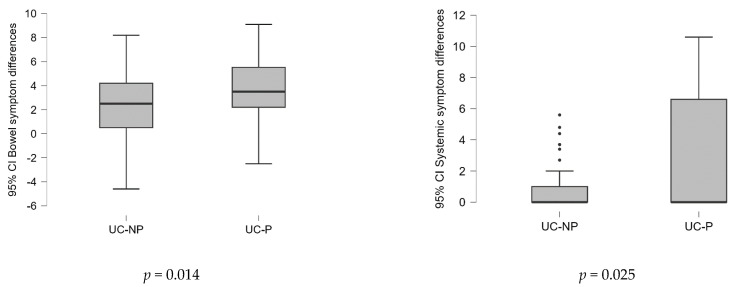

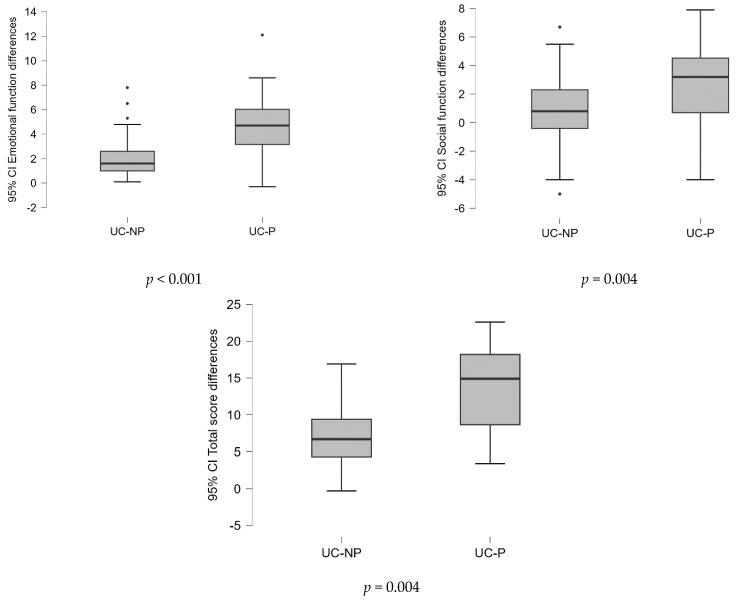
Differences of the values of variables in the two time points (before/after the treatment) analyzed between samples on SIBDQ score (Mann–Whitney U test).

Testing the differences between the average scores by domain and total, at the two evaluated time points (before vs. after the treatment) between samples (Figure 2), the results showed that the probiotics associated with L-glutamine and biotin in diet strategy were substantially more effective (*p* < 0.05). 

**Figure 3:**
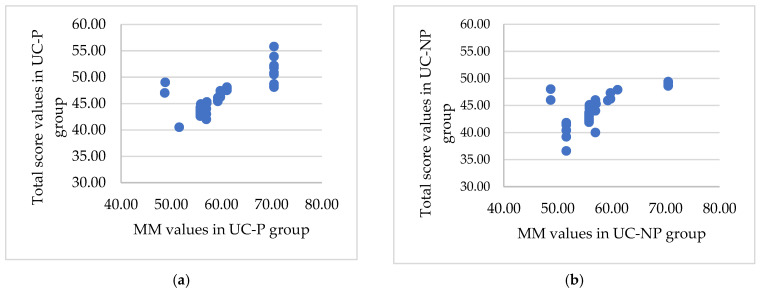

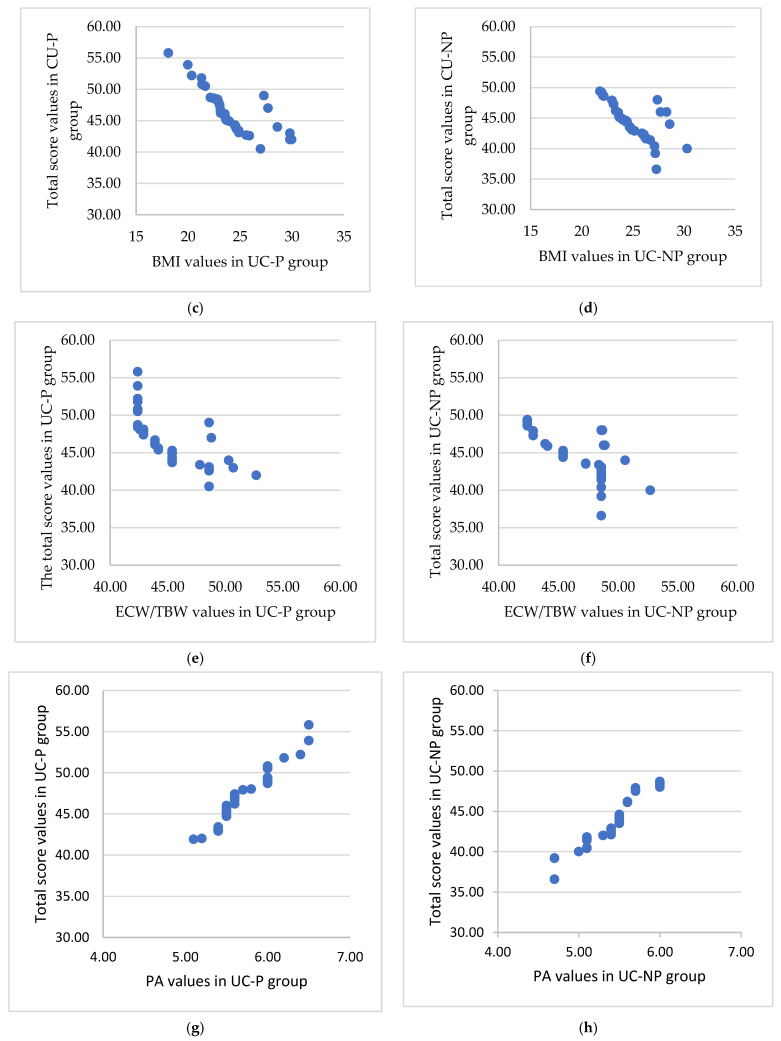
The significant association between the total score and the muscle mass, BMI, ratio ECW/TBW, and PA for the UC-P (**a**,**c**,**e**,**g**) and UC-NP group (**b**,**d**,**f**,**h**). (**a**) Strong positive significant association (r=0.75,p<0.001) between total score and muscle mass. (**b**) Medium positive significant (r=0.64,p<0.001) association between total score and muscle mass. (**c**) Strong negative significant association between total score and BMI (UC-P group). (**d**) Medium negative significant association $\left(r=-0.65,p<0.001\right)$ between total score and BMI (UC-NP group). (**e**) Strong negative significant association (r=−0.77,p<0.001) between total score and ratio ECW/TBW. (**f**) Strong negative significant association $\left(r=-0.75,p<0.001\right)$ between total score and ratio ECW/TBW. (**g**) Very strong positive significant association (r=0.97, p<0.001) between total score and PA. (**h**) Very strong positive significant association (r = 0.96, *p* < 0.001) between total score and PA.

The SIBDQ total score was used as a response in an exhaustive correlation analysis that evaluated the relationships between quality of life and the identified physical indicators. The results showed a strong correlation in four instances $\left(\left|r\right|>0.5,p<0.001\right)$. The group of patients that take probiotics had better overall results (Figure 3).

## 3. Discussion

The current study was designed to assess the nutritional status and body composition of UC patients, utilizing bioelectrical impedance analysis (BIA), and to examine the clinical effects of a dietary-based probiotics supplementation on body composition variations and quality of life. Although the positive influence of probiotics on body parameters in different health conditions was highlighted in some studies [33,34,35], as far as we are aware, this is the first UC patient study to evaluate the impact of a supplement with probiotics on body composition. Researchers’ interest in IBD has grown recently, but recent publications have tended to be more treatment- and mechanism-oriented, and they do not seem to take into account the nutritional status of IBD patients [36]. 

Because of the UC’s characteristics, in addition to medication and surgery, dietary and nutritional counseling is a crucial component of treatment that, despite its importance, it is sometimes overlooked in clinical practice [37,38]. Even though there is not any precise dietary recommendation for IBD, more than 70% of patients report that poor nutrition has a substantial impact on the evolution of the condition and worsens the intensity and frequency of symptoms [38,39,40]. In order to promote a healthy course of the disease and improve the quality of life for UC patients, nutritional strategies should be tailored to the individual needs of each patient [41].

In our study population, most of the UC patients had excessive body mass, meaning that their BMI was above the acceptable level. Recently published studies showed that a significant number of patients had considerable body obesity [42,43,44]. Despite the fact that their diets may contain enough calories, it has been reported that people with UC may not consume enough nutrients, regardless of how severe their disease is [45]. This may lead to nutrient shortages without a decrease in BMI. 

Results from earlier research indicated that UC patients frequently lost skeletal muscle mass, which was more sensitive to changes in BMI [46,47]. An early diagnosis of skeletal muscle loss and malnutrition in UC patients could be made using body composition measurement, which could also help direct rapid therapeutic nutrition management [46].

Increasing evidence from recent years supports the theory of a “gut–muscle axis”, according to which starvation, inflammation, and gut dysbiosis interact to cause muscle failure in IBD subjects [48]. Probiotics can be administered as a helpful tool in the management of UC treatment. Recent research on the mechanism of action of probiotics has revealed that these organisms can directly affect intestinal health and epithelial cell function by modifying epithelial cytokine secretion into an anti-inflammatory dominant profile [49]. 

According to some studies, several probiotic strains, like different *Lactobacillus* and *Bifidobacterium* species, may improve UC [50,51]. 

The dietary supplement used for the UC patients in this study was a combination of five bacterial strains of at least five billion bacteria (*Bifidobacterium infantis*, *Bifidobacterium animalis*, *Lactobacillus bulgaricus*, *Lactobacillus helveticus*, and *Enterococcus faecium*) with L-glutamine and biotin, two nutrients whose administration has been found to be beneficial for individuals with symptoms of intestinal inflammation. The supplement was administrated over the course of three consecutive treatment sessions, separated by 12-week breaks. This therapy strategy had a positive impact on the weight status and body composition indicators in UC patients. The average value of MM and SMI at the level of muscle parameters significantly increased (*p* < 0.001) and a large fraction of patients had their SMI levels normalized. Even if at the MM level there was a significant increase in the control group, indicating the benefits of nutritional counseling, at the SMI level the changes were not significant. According to the findings of a recent systematic analysis of randomized clinical trials examining the impact of probiotics on muscle growth, total lean mass, and muscle strength in young and older persons with different health conditions, probiotic supplementation increases both muscle mass and overall muscle strength [33]. SMI is a crucial marker for determining how muscular a person is. Poor SMI, frequently seen in UC patients, is a sign of sarcopenia [52], a condition that is typically brought on by a variety of factors, such as inactivity, endocrine dysfunction, chronic illnesses, systemic inflammation, and malnutrition [53]. In a Chinese study, data on 80 patients with active UC and 80 healthy participants who were age- and sex-matched were gathered. In comparison to the healthy control group, the incidence of low SMI and malnutrition was much higher in the UC group (*p* <0.05); 62.5% of UC patients with a normal body mass index had low SMI [46]. The results suggested that skeletal muscle loss is more common in UC patients and more susceptible than changes in BMI. To identify and treat UC patients who are at a high risk for malnutrition, clinicians should measure the loss of skeletal muscle mass in addition to changes in BMI. 

The ECW/TBW ratio, in which a significant drop was noted in the studied group (UC-P) compared to the control group (UC-NP), also had significantly different mean values (*p* = 0.022). The analysis of the ECW/TBW ratio evolution in each of the two groups showed insignificant changes in the control group (*p* = 0.783) and significant changes in the study group (*p* = 0.015), indicating the positive impact of the tested supplement. Other studies reached the conclusion that ECW/TBW is an important indicator of how well-balanced the body’s water is. High ECW/TBW can be a sign of edema, swelling, or inflammation, whereas lower ECW/TBW is often seen in those who have greater levels of SMM [54,55]. 

Unfortunately, there is little information available on the body composition of individuals who have inflammatory bowel disease (IBD). Changes in fat and muscle may have an impact on overall morbidity, quality of life (QoL), muscle function, and bone health. Even if the findings are varied, many UC patients appear to have abnormalities in their lean and fat mass, which may not be seen during conventional clinical examinations [56]. 

Adipose tissue is pro-inflammatory. Fat-free mass (FFM) includes body water, skeletal and smooth muscle mass, and bones [57]. It has been demonstrated that increasing fat-free mass can lessen the adverse effects of fat mass. The ratio of fat mass to fat-free mass (FM/FFM) has been linked to other chronic diseases such as cardio-metabolic diseases and non-alcoholic fatty liver disease, and to unfavorable outcomes, according to a number of recent research [58,59]. In the UC population that was being researched, a large proportion of individuals with excessive FM and a low FFM share were associated with the indicated excessive body mass. A study conducted at the Department of Dietetics, Warsaw University of Life Sciences, which included 44 individuals with UC in remission, observed higher values for BMI, FM, and FFM [60]. Body composition, including FM and FFM, plays an important role in the diagnosis and management of sarcopenia. Muscle quality was only recently added to the definition, in 2019 [61]. 

Our study tracked the progression of the patients in the two groups, measuring the difference between the parameter values at the initial and final moments to determine the efficacy of one therapy approach in comparison to the other. Statistically significant differences in favor of the group with probiotics associated with L-glutamine and biotin were observed, both at the level of weight status (BMI) and at the level of FFM and ECW/TBW parameters.

The results of this study showed that the BMI, SMI, FM, and PA evolution of the patients in comparison to the reference values was substantially improved (*p* < 0.05) only in the UC-P group. In IBD patients, PA is considered a marker that helps in evaluating nutritional status [62]. Low PA values are linked to apoptosis or modification in the selective permeability of membranes, endangering their integrity and metabolic activities, since it is thought to be a major predictor of health state, including malnutrition, inflammation, and disease [63,64,65,66]. A good correlation was shown between high PA value and preserved skeletal muscle structure in terms of volume and/or functionality [62].

Also, the findings of this study revealed that patients in the UC-P group had considerably lower CRP values than patients in the UC-NP group. These results are consistent with previous reports, in which the volume of skeletal muscle was closely correlated with C-reactive protein levels [46]. CRP is a significant marker of systemic inflammation that is widely used in clinical settings to assess the degree of intestinal mucosa damage. Ideal serum indicators for IBD are still not available. Combining various biomarkers can help improve disease evaluation performance [67]. Despite the fact that high levels of CRP can be linked to diseases other than IBD, data from a recent study suggest that CRP more strongly represents colon-wide mucosal inflammation than fecal calprotectin and provides a reliable assessment of inflammation across the colon in individuals with active UC [68]. 

UC has an impact on the health-related quality of life (HRQoL). Reduced HRQoL has been documented even in silent UC, despite the fact that disease activity is the most significant factor. It is believed that sociodemographic, clinical, psychological, and treatment-related factors influence HRQoL in IBD patients [69]. 

In this investigation, when the associations between changes in physical parameters and the quality-of-life score determined by the SIBDQs were tested, the results showed stronger relationships between the variables in the UC-P group than in the UC-NP group, which led to the conclusion that probiotics came with significant benefits in terms of maintaining body composition. 

Our study has the advantage of being one of the few to date that has looked at the relationship between body composition and dietary supplementation with probiotics in UC patients. This research does, however, have certain drawbacks. First, there were not many patients included and we are aware that there are other significant variables that could affect body composition, such as illness activity and duration or glucocorticoid exposure, which have not been explored. Other limitations of this study include the fact that adherence to therapy was not assessed using specific indicators and that CRP was the only pro-inflammatory biomarker considered. Despite these limitations, our results indicate a significant correlation between the consumption of such dietary supplements and the positive evolution of body composition parameters and improvements in the subjects’ quality of life. However, future research is needed to avoid the previously mentioned limitations, as well as to support/validate the results of this study.

## 4. Materials and Methods

### 4.1. Patients’ Selection

**Figure 4:**
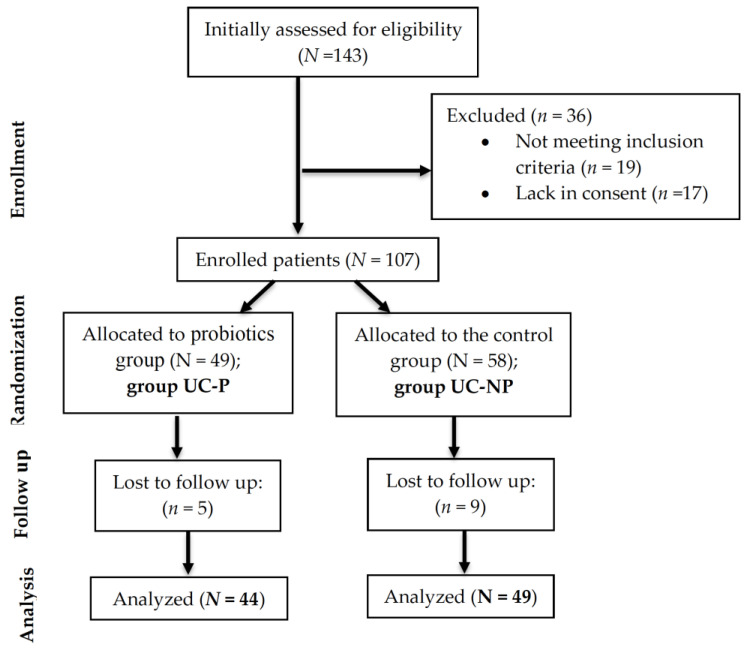
CONSORT flow diagram of the study.

A total of 93 patients with a confirmed diagnosis of UC effectively participated in this observational follow-up randomized study, which was conducted between January 2022 and January 2023 in the Pharmacy Department of the University of Oradea, Oradea, Romania, in collaboration with a private dietetics’ office from the same town. Initially, the recruitment involved 143 consecutive UC patients aged ≥18 years from the specialist outpatients of the Bihor County Emergency Clinical Hospital and private clinics located in Oradea, Romania. The subjects included in the study had to meet diagnostic standards determined by clinical, laboratory, imaging, and endoscopic parameters, including histopathology, in accordance with the 2017 ECCO recommendations [66]. Subjects over 18 years of age, for whom body composition could be accurately determined, were selected. Patients with ascites and severe edema, those with oncological, musculo-skeletal or psychiatric pathologies, and those who took probiotics supplements in the last 6 months were excluded. After applying the inclusion/exclusion criteria, only 107 patients were enrolled in the study. Among them, 14 did not show up for all the evaluations and were excluded from the analysis (Figure 4). All the included patients were on treatment, recommended by a specialist. 

At the time of recruitment, the participants had the options of receiving nutritional counseling and body composition/assessment and using of standardized nutritional supplement developed by HLH Biopharma (Balve, Germany) for diet management of inflamed intestinal mucosa, which is available online on the producer’s site [70]. Investigators allocated an adequate time for each recruited patient who was interested in the study, to explain the importance of investigating nutritional support and body composition and to present the possible benefits of the recommended supplement. 

### 4.2. Study Design

The objective of this follow-up randomized study was to evaluate the impact on body composition and quality of life of a standardized nutritional supplement in the form of enteric-coated capsules containing five bacterial strains of at least five billion bacteria (*Bifidobacterium infantis*, *Bifidobacterium animalis*, *Lactobacillus bulgaricus*, *Lactobacillus helveticus*, and *Enterococcus faecium*), L-glutamine, and biotin—developed for diet management of inflamed intestinal mucosa [65]. Patients were randomly split into two groups: UC-P (44 patients with dietary counselling and the supplement with probiotics associated with L-glutamine and biotin) and UC-NP (49 patients with dietary counselling, without supplement with probiotics associated with L-glutamine and biotin). The supplement was given to patients in the UC-P group in 3 sessions of 4 weeks each (2 tablets per day, regardless of meals), with 12-week breaks in between (the evaluation/follow-up period of a patient from the initial moment of inclusion in the study to the final one, totaling 36 weeks, as follows: 4 weeks of treatment × 3 sessions = 12 weeks of treatment, plus 2 more rest periods of 12 weeks each between each treatment session—respectively, 24 weeks).

The short inflammatory bowel disease questionnaires (SIBDQs) score and changes in body composition were used as the basis for the evaluation. A nutrition professional assessed the nutritional status and body composition of each patient, and the patients were given tailored nutritional advice based on their symptoms, comorbidities, and distinctive physiological traits. The dietary recommendations were created generally to provide a balanced intake of nutrients and energy. The diet consisted of a moderate quantity of soft fiber-rich carbohydrates, lean animal proteins free of fat, without sausages or other highly processed or spicy foods, and a small amount of fat (ideally from vegetables rich in unsaturated fatty acids). Boiling or baking was the recommended method of food preparation, including fruit, in order to prevent the fermentation process.

All patients were evaluated monthly from a nutritional point of view (with BIA) to check their adherence and response to diet and therapy. The final evaluation was carried out at the conclusion of the last treatment for the UC-P group and at the conclusion of the study for the UC-NP group. The initial results and those from the last evaluation were statistically analyzed comparatively between the groups.

C-reactive protein (CRP) was evaluated as an inflammatory marker at the beginning and at the end of the study. The most frequent serum biomarker of inflammation in IBD is CRP. CRP levels that are higher help in separating latent IBD from mucosal active illness. IBD is in remission when the CRP level is under 10 mg/L [71]. 

### 4.3. Body Composition Measurement

The measurement of nutritional status and body composition was done using the Tanita Corporation’s multifrequency bioelectrical impedance device (MF-BIA) with eight electrodes, model number MC780MA (Tanita Corporation, Tokyo, Japan). The method involves determining the body resistance when an electric current, of low intensity, travels through it at different frequencies (5 kHz, 50 kHz, and 250 kHz) [72]. The BIA measurements were performed by a qualified team in accordance with established protocols. The participants were asked to stand with bare feet on the metal foot plate of the analyzer, gently hold the hand grip with their arms straight and hanging down in a neutral standing position, avoid skin-to-skin contact, and undergo an overnight fast. The ambient temperature was kept at 25 °C. The following parameters were assessed for each participant in the study: body mass index (BMI), skeletal muscle mass index (SMI), fat-free mass (FFM), muscle mass (MM), skeletal muscle mass (SMM), fat mass (FM), ratio of extracellular body water/total body water (EBW/TBW), and phase angle (PA). The WHO guidelines were used to evaluate the BMI [73] (<18.5 kg/m^2^—malnutrition; <18.5; 25 kg/m^2^—appropriate body mass; <25 to 30 kg/m^2^—overweight; and ≥30 kg/m^2^—obesity). There are reference values based on gender, age, weight, and height for some of the parameters evaluated, and these were evaluated in accordance with the manufacturer’s recommendations (Tanita Corporation, Tokyo, Japan): SMI normal values > 5.76 for women and > 7 for males, FM% normal values 23–34%, and PA normal values > 5.5.

### 4.4. Short Inflammatory Bowel Disease Questionnaire (SIBDQ)

The Short Inflammatory Bowel Disease Questionnaire, a 10-item self-reporting evaluation on the implications of respondents’ IBD (here, UC) on the health-related quality of life (HRQoL) was used to assess disease-specific HRQoL [74]. The SIBDQ has been demonstrated to be valid, consistent, and responsive for evaluating disease specific HRQoL in groups of UC patients [75,76]. Bowel symptoms, social function, systemic symptoms, and emotional function are the four categories that the SIBDQ assesses. A cumulative score is determined from the answers to all parameters. Values for the three-item bowel symptoms and the two-item emotional function fields scores vary from 3 to 21 points, while the two-item systemic symptoms and social function fields’ scores vary from 2 to 14 points. The total score may range from 10 to 70 points. Higher values correspond to improved HRQoL across all fields and the overall score. When the HRQoL reached 10–45 points, it was deemed to be significantly degraded; it was moderately impaired at 45–60 points and mildly impaired at 60–70 points [77].

### 4.5. Statistical Analysis

For gathering the data, Microsoft Excel 2021 was used, and for the statistical analysis SPSS 20 (New York, NY, USA), JASP 17.1 (University of Amsterdam, Department of Psychology and Psychological Methods Unit, Amsterdam, the Netherlands) and Microsoft Excel were used. Data are given as means, standard deviations (SDs), and minimum, maximum, and median values in descriptive statistics. The Shapiro–Wilk test was used to confirm the distribution. The Mann–Whitney U test (for nonparametric distribution) and the chi^2^ test (for categorical data) were both applied to the research to test for variations in BMI and body composition parameters between groups. The total score of the SIBDQ scale served as a predictor in a correlation model that was used in order to identify any potential relationships between the variables under consideration. The level of significance was set at *α* = 0.05. Given the total number of patients (N = 143) considered for our research, the sample size could be estimated, resulting in 77 subjects being the representative sample size, by using the OpenEpi software, version 3.01. [78] and defining the power of the test at 80%. 

## 5. Conclusions

Analyzing the results obtained, it can be suggested that the consumption of dietary probiotics associated with L-glutamine and biotin can improve body composition parameters, which in turn implies an increase in the overall quality of life of patients with UC. Larger scale clinical trials are needed to validate this conclusion and to optimize the outcomes. 

## Figures and Tables

**Table 1:** Baseline characteristics of the groups.

Parameter	UC-NP	UC-P	*p* ^1,2^
N	49	44	0.322 ^1^
Age, M, SD	44.20 ± 14.86	45.53 ± 12.49	0.647 ^2^
Range of age (min–max)	58 (18–76)	59 (20–79)	-
Female, N (%)	22 (45.1)	27 (61.4)	0.731 ^1^
Urban area, N (%)	31(63.2)	21 (44.7)	0.430 ^1^
BMI, M, SD	25.45 ± 2.54	26.28 ± 2.56	0.122 ^2^
Smoker, N (%)	14 (28.6)	12 (27.3)	0.319 ^1^
Alcohol user, N (%)	9 (18.4)	5 (11.4)	0.220 ^1^
FH IBD, N (%)	17 (34.7)	8 (18.2)	0.928 ^1^
CPR < 10 (mg/L), N (%)	30 (61)	22 (50.0)	0.854 ^1^

M—mean value; SD—standard deviation value; N—number of the patients; BMI—body mass index; CRP—C-reactive protein, UC-P—group with dietary counselling and supplement with probiotics, L-glutamine, and biotin; UC-NP—group with dietary counselling, without supplement; ^1^—*p* values given by the chi-square test, ^2^—*p* values given by the Mann–Whitney U test.

**Table 2:** Descriptive statistics of the samples displayed throughout the two time periods examined.

Parameters	UC-NP				UC-P				*p* ^1^
	95% CE Mean ± 2 × SD	Median	Min.	Max.	95% CE Mean ± 2 × SD	Median	Min.	Max.	
Baseline									
BMI (kg/m^2^)	25.45 ± 5.08	25.50	19.36	30.62	26.28 ± 5.12	26.10	19.32	31.74	0.122
SMI (kg/m^2^)	7.15 ± 1.74	7.62	6.03	8.81	7.50 ± 1.76	9.50	6.04	9.98	0.059
FFM (kg)	54.08 ± 14.28	51.60	45.10	74.20	54.65 ± 17.08	51.60	45.10	74.20	0.730
MM (kg)	48.90 ± 16.64	46.30	42.10	70.50	50.16 ± 18.74	46.30	42.10	70.50	0.494
SMM (kg)	28.73 ± 10.06	29.20	21.20	39.80	28.85 ± 11.56	29.60	21.20	39.80	0.912
FM (%)	32.40 ± 9.34	32.10	27.90	43.50	30.92 ± 8.44	27.90	27.90	48.70	0.116
PA (^0^)	5.44 ± 0.54	5.30	5.00	6.10	5.40 ± 0.52	5.30	4.90	5.90	0.433
ECW/TBW (%)	45.09 ± 6.30	43.00 *	42.90	55.50	45.17 ± 5.88	43.30	43.00	55.50	0.084
Follow-up									
BMI (kg/m^2^)	24.39 ± 4.76	23.68	19.99	30.30	24.78 ± 4.34	24.55	18.11	29.97	0.421
SMI (kg/m^2^)	7.32 ± 1.21	6.98	5.97	9.98	8.37 ± 1.28	8.93	6.04	9.96	0.001 *
FFM (kg)	55.52 ± 17.9	51.60	44.00	74.20	58.76 ± 21.34	55.80	45.10	76.00	0.113
MM (kg)	58.00 ± 13.7	55.90	48.70	74.20	60.82 ± 15.50	57.00	48.70	74.20	0.043 *
SMM (kg)	34.51 ± 11.02	34.60	22.50	40.60	33.43 ± 12.12	32.60	20.80	40.40	0.371
FM (%)	29.94 ± 9.82	28.20	25.20	43.30	28.51 ± 9.38	25.20	25.20	45.20	0.156
PA (^0^)	5.59 ± 0.56	5.50	5.10	6.50	5.55 ± 0.48	5.50	5.00	6.00	0.529
ECW/TBW (%)	44.71 ± 5.84	42.90	42.30	52.70	43.50 ± 3.78	42.40	42.40	48.60	0.022 *

BMI—body mass index; SMI—sarcopenic index; FFM—fat-free mass; MM—muscular mass; SMM—skeletal muscle mass; FM—fat mass; PA—phase angle; ECW/TBW—ratio of extracellular water to total body water; CE—confidence interval; ^1^—value given by the Mann–Whitney U test; UC-P—group with dietary counselling and supplement with probiotics, L-glutamine, and biotin; UC-NP—group with dietary counselling, without supplement; *—significant values.

**Table 3:** The data dynamics of the samples displayed throughout the two time periods examined.

Parameters	UC-NP			UC-P		
	Statistic	z-Score	*p* ^1^ Value	Statistic	z-Score	*p* ^1^ Value
BMI (kg/m^2^)—B vs. F	959.00	3.805	<0.001 *	816.00	4.995	<0.001 *
SMI (kg/m^2^)—B vs. F	517.5	−0.251	0.806	214.00	−3.279	0.001 *
FFM (kg)—B vs. F	229.5	−1.841	0.067	0.00	−4.197	<0.001 *
MM (kg)—B vs. F	6.00	−5.969	<0.001 *	1.00	−5.765	<0.001 *
SMM (kg)—B vs. F	0.00	−6.093	<0.001 *	22.00	−5.37	<0.001 *
FM (%)– B vs. F	1225.00	6.023	<0.001 *	990.00	5.777	<0.001 *
PA (^0^)—B vs. F	32.00	−5.774	<0.001 *	114.00	−4.446	<0.001 *
ECW/TBW (%)—B vs. F	519.00	0.280	0.783	335.00	−2.423	0.015 *

B—baseline; F—follow-up, BMI—body mass index; SMI—sarcopenic index; FFM—fat-free mass; MM—muscular mass; SMM—skeletal muscle mass; FM—fat mass; PA—phase angle; ECW/TBW—ratio of extracellular water to total body water; ^1^—*p* value given by the Wilcoxon signed rank test; *—significant values; static—statistic of Wilcoxon signed rank test; z-score—statistic measurement describing the relationship between a value and the mean of a group/set of values.

**Table 4:** BMI, body composition, and CRP in comparison with reference values—number of individuals characterized by values in categories between groups.

References Values/ Time Moments		UC-NP (N = 49)		UC-P (N = 44)		
		N	%	N	%	*p* ^1^
BMI (kg/m^2^)						
Normal weight	B	21	42.8	14	31.8	0.001 *
	F	31	63.3	29	65.9	0.001 *
Overweight	B	26	53.1	27	61.4	0.001 *
	F	17	34.7	15	31.8	0.001 *
Obese	B	2	4.1	3	6.8	0.510
	F	1	2.04	-		-
SMI (kg/m^2^)						
Low	B	12	24.5	6	13.6	0.157
	F	16	32.7	2	4.5	0.163
Normal	B	37	75.5	38	86.4	0.908
	F	33	67.3	42	95.5	0.001 *
FM (%)						
Normal	B	28	57.1	28	63.6	0.101
	F	32	65.3	33	75.0	0.010 *
Over	B	21	42.9	16	36.4	0.386
	F	17	38.7	11	25.0	0.001 *
PA (^0^)						
Under	B	47	95.9	44	100	0.346
	F	14	28.6	8	18.2	0.002 *
Normal	B	2	4.1	-	-	0.749
	F	35	71.4	36	81.8	0.001 *
CRP (mg/L)						
<10 mg/L	B	30	61.2	22	50.0	0.116
	F	37	75.5	40	90.9	1.000
>10 mg/L	B	19	38.8	22	50.0	0.001 *
	F	12	24.5	4	81.8	0.001 *

^1^—*p* value given by the chi^2^ test; *—significant values; BMI—body mass index; SMI—sarcopenic index; FM—fat mass; PA—phase angle; UC-P—group with dietary counselling and supplement with probiotics; UC-NP—group with dietary counselling, without supplement with probiotics; B—baseline; F—follow-up.

**Table 5:** Evolution of the SIBDQ score resulting from the Mann–Whitney U test between the two groups.

SIBDQ Domains		UC-NP (N = 49)	UC-P (N = 44)		
		95% CE Mean ± 2 × SD	95% CE Mean ± 2 × SD	t	*p* ^1^
Bowel symptoms	B	13.11 ± 3.94	13.39 ± 2.78	0.625	0.431
	F	15.38 ± 4.74	17.05 ± 3.44	14.771	<0.001 *
Systemic symptoms	B	9.24 ± 2.88	9.33 ± 2.80	0.086	0.770
	F	10.03 ± 3.04	12.14 ± 6.80	15.498	<0.001 *
Emotional function	B	13.50 ± 2.94	14.03 ± 3.34	2.574	0.112
	F	15.62 ± 2.62	18.85 ± 2.62	104.076	<0.001 *
Social function	B	9.08 ± 2.98	9.83 ± 2.98	3.738	0.056
	F	10.02 ± 3.68	12.43 ± 3.68	36.330	<0.001 *
Total score	B	45.21 ± 5.64	45.58 ± 5.64	3.524	0.061
	F	51.05 ± 6.54	60.47 ± 6.54	155.871	<0.001 *

^1^—Mann–Whitney U test; *—significant values; UC-P—group with dietary counselling and supplement with probiotics; UC-NP—group with dietary counselling, without supplement with probiotics; B—baseline, F—follow-up.

**Table 6:** Evolution of the SIBDQ score resulting from the Wilcoxon signed rank test between the two time moments.

Parameters	UC-NP			UC-P		
	Statistic	z-Score	*p* ^1^ Value	Statistic	z-Score	*p* ^1^ Value
Bowel symptoms—B vs. F	117.00	−4.730	<0.001 *	17.00	−5.578	<0.001 *
Systemic symptoms—B vs. F	0.00	−3.516	<0.001 *	0.00	−3.920	<0.001 *
Emotional function—B vs. F	0.00	−6.093	<0.001 *	1.00	−5.765	<0.001 *
Social function—B vs. F	330.5	−2.641	0.008 *	109.5	−4.389	<0.001 *
Total score—B vs. F	3.00	−6.063	<0.001 *	0.00	−5.777	<0.001 *

B—baseline; F—follow-up, ^1^—Wilcoxon signed rank test; *—significant values.

## Data Availability

Patients’ data are available by request from the first author’s archive.
